# Special Issue on Differential Gene Expression and Coexpression (2nd Edition)

**DOI:** 10.3390/biology15151231

**Published:** 2026-07-23

**Authors:** Apostolos Malatras, Ioannis Michalopoulos

**Affiliations:** 1 Biobank.cy Centre of Excellence in Biobanking and Biomedical Research, University of Cyprus, 2029 Nicosia, Cyprus; 2 Centre of Systems Biology, Biomedical Research Foundation, Academy of Athens, 11527 Athens, Greece

Transcriptomics refers to the study of all transcripts of a cell or a tissue, as well as transcript expression quantification. Transcriptomics technologies include microarrays and RNA-Seq. The accumulation of transcriptomics samples freely available in public repositories has enabled the massive re-analysis of those data, which transcends the scope of the original experiments. The main transcriptomics approach is differential gene expression analysis (DGEA), where comparison between samples of different conditions is performed, leading to the discovery of differentially expressed genes (DEGs) which may be responsible for phenotype differences between these biological conditions. An alternative approach is gene coexpression analysis which identifies groups of genes with similar expression patterns across unrelated sets of transcriptomics data of the same organism from the same experimental platform. Coexpressed genes tend to be involved in similar biological processes and pathways [1].

In the original version of the Special Issue on “Differential Gene Expression and Coexpression”, the central roles of differential expression and gene coexpression analyses in deciphering phenotypic diversity and functional molecular networks were established [2]. In Special Issue “Differential Gene Expression and Coexpression (2nd Edition)”, these methodologies were expanded at the intersection of advanced computational pipelines, systemic benchmarking, and functional biology across various eukaryotic systems. It comprises six research articles offering user-friendly publicly available software, novel mechanistic insights, as well as advances in transcriptomics analysis. By bridging computational development with experimental and bibliographical validation, these studies showcase how primary transcriptomics data may be translated into systems-level models and uncover disease biomarkers, therapeutic targets, and evolutionary stress-tolerance mechanisms.

Regarding microarray-based Differential Gene Expression Analysis, Katsiki et al. [3] introduce DExplore, an intuitive web application, which also includes a Docker image installation, that simplifies differential expression analysis for Affymetrix microarrays, while integrating functional enrichment. DExplore enables non-technical wet-lab researchers to perform high-throughput-based DGEA, as well as downstream enrichment analysis of the produced DEG lists. Dermitzaki et al. [4] conducted a microarray-based gene expression meta-analysis of Alzheimer’s disease brain samples. After an extensive search in public microarray databases, ten sub-studies were combined using the Mosteller–Bush approach, identifying a total of 4218 DEGs. These DEGs showcase a transcriptomic imbalance characterised by up-regulated genes related to immune, cytokine, and inflammatory pathways compared to the down-regulation of genes related to synaptic vesicles, neurotransmission, and mitochondrial oxidative phosphorylation.

Moving to RNA-Sequencing, Kozlova et al. [5] compared the alternative splicing profiles of normal liver tissue and malignant hepatocyte cell lines (HepG2 and Huh7). By presenting splicing profiles as arrays of genes characterised by their “degree of alternative splicing” (DAS) which is the number of detected splice variants of each gene, the authors demonstrated that this metric can successfully identify altered biological pathways using gene set enrichment analysis (GSEA), revealing that individual splice variant abundances offer greater phenotypic specificity than integral gene expression profiles. To address the lack of standardisation in bulk RNA-Seq workflows, Carels [6] employed network-level Shannon entropy of up-regulated malignant genes as an objective metric to benchmark several RNA-Seq preprocessing pipelines. By investigating twelve distinct cancer types, this study revealed that non-parametric normalisation methods, specifically TPM coupled with a log_2_ fold-change filter, are superior in preserving the negative correlation between tumour network complexity and patient survivability compared to complex parametric tools.

Experimentally validating bioinformatics outputs, Lee et al. [7] investigated mucosal barrier disruption and intestinal inflammation in *Drosophila melanogaster* following oral DSS administration. Using transcription factor enrichment tools, the authors initially screened genes related to mucus disruption and eventually identified *GlcAT-S* experimentally as a main enterocyte-expressed regulator. This gene’s knockdown severely compromised gut length, triggering compensatory stem cell proliferation, shutting down key mucin genes, and greatly amplifying the expression of inflammatory cytokines. Thus, the possibility of *GlcAT-S* and its corresponding human ortholog (*B3GAT3*) serving as targets for the treatment of intestinal inflammatory diseases was suggested. Finally, Li et al. [8] presented the genome-wide identification of the thioredoxin system in the selenium hyperaccumulator *Cardamine hupingshanensis*, mapping 74 typical and atypical *ChTRX* genes along with 12 *ChTR* genes. Their work highlighted a prominent chloroplast-localised thioredoxin expansion, utilising molecular docking to predict that the stress-responsive *ChACHT4-1* dynamically regulates the redox states of key selenium metabolism enzymes (*ChAPK* and *ChAPR*), integrating stress tolerance and primary assimilation pathways.

In conclusion, the works included in the second edition of this Special Issue showcase the evolution of transcriptomics into an integrated, systems-level discipline. We are confident that the resources, pipelines, and biological discoveries presented here will serve as a valuable reference for future research in biomarker discovery, personalised medicine, and biotechnology. The guest editors are grateful to all authors for their contributions to the “Differential Gene Expression and Coexpression (2nd edition)” special issue. We are looking forward to the submission of novel bioinformatics works and biological findings in the next edition of the special issue: “Differential Gene Expression and Coexpression (3rd Edition)”.
